# The 50% effective dose of hydromorphone and morphine for epidural analgesia in the hemorrhoidectomy: a double-blind, sequential dose-finding study

**DOI:** 10.1186/s12871-024-02420-0

**Published:** 2024-01-30

**Authors:** Xianghua Cao, Qiangjun Gui, Yujiao Wei, Lanhui Lan, Huiling Xiao, Shihong Wen, Xueping Li

**Affiliations:** 1 Department of Anesthesiology, Dongguan Tungwah Hospital, Dongguan, China; 2 Department of Anesthesiology, The First Affiliated Hospital, Sun Yat-Sen University, No 58, ZhongShan 2nd road, Guangzhou, China

**Keywords:** ED50, Hydromorphone, Morphine, Epidural analgesia, Hemorrhoidectomy, Randomized controlled trial

## Abstract

**Background:**

Although previous studies have showed that epidural morphine can be used as a complement to local anesthetics for analgesia after postcesarean delivery under spinal anesthesia, there is little known about the analgesic dosage of epidural morphine and hydromorphone for hemorrhoidectomy. Therefore, we conducted this study to examine the potency ratio of hydromorphone to epidural morphine as well as effective analgesic dose for 50% patients (ED50) undergoing elective hemorrhoidectomy.

**Methods:**

80 patients under elective hemorrhoidectomy with combined spinal and epidural anesthesia(CSEA) in department of anesthesia, Dongguan Tungwah hospital. To assess the ED50, patients were treated with epidural morphine or epidural hydromorphone randomly using a biased coin method-determined dose with a sequential allocation procedure. Following surgery, standardized multimodal analgesia was administered to all patients. A pain response score of ≤ 3 (on a scale of 0–10) was determined to be the effective dose after 24 h following CSEA. The ED50 in both groups were determined using the probit regression and isotonic regression method. We also measured pain intensity by patient interview using a 10 point verbal numeric rating scale prospectively at 6, 12 and 24 h after CSEA, and adverse effects were also noted.

**Results:**

The ED50 was 0.350 mg (95% CI, 0.259–0.376 mg) in hydromorphone group and 1.129 mg (95% CI, 0.903–1.187 mg) in morphine group, respectively, estimated by isotonic regression method. Regression analysis with the probit, the ED50 of epidural hydromorphone was 0.366 mg (95% CI, 0.276–0.388 mg) and epidural morphine was 1.138 mg (95% CI, 0.910–1.201 mg). Exploratory findings showed that there was no difference between the most frequent dosages of epidural hydromorphone or epidural morphine in the occurrence of nausea, vomiting and pruritus. When administered with epidural opioids at ED50 doses or higher, 97.5% (39/40) of epidural morphine patients and 97.5% (39/40) epidural hydromorphone of patients were satisfied with their analgesia.

**Conclusion:**

Effective hemorrhoidectomy analgesia requires a 3:1 ratio of epidural morphine to epidural hydromorphone. Both drugs provide excellent patient satisfaction.

**Supplementary Information:**

The online version contains supplementary material available at 10.1186/s12871-024-02420-0.

## Introduction

In the United States, hemorrhoids was estimated to affect 10 million people annually, with an incidence of 4.4%. Over 37 patients out of every 100,000 people underwent hemorrhoidectomies each year [1]. A significant clinical issue in this approach is severe postoperative discomfort following hemorrhoidectomy. For patients who have undergone a hemorrhoidectomy, effective postoperative pain management is crucial to promoting healing and reducing hospital stays [2–4].

Contrary to prevalent trends, less administration of opioids during surgery may have some adverse effects, such as more postoperative pain and increased opioid usage, and maximized usage of delivery opioids during surgery may improve the long-term outcomes [5]. Thus, opioids, acetaminophen, nonsteroidal anti-inflammatory medications, peripheral nerve blocks, neuraxial blocks (epidural and paravertebral), and local anesthetic infiltration are among the multimodal analgesia methods now used for postoperative pain control [6]. To our knowledge, intrathecal morphine and hydromorphone are beneficial when used in a multimodal regimen for postcesarean delivery analgesia [7–10]. However, managing perioperative pain remains a major challenge, impeding postoperative recovery [11]. Until now there is a paucity of information on its effectiveness in hemorrhoidectomy anesthesia in the epidural space.

Effective perioperative pain management strategies remain an anesthesiologic challenge [12]. Epidural morphine and hydromorphone of effective analgesic dose for 50% patients for hemorrhoidectomy analgesia has not yet been fully determined by any previous prospective research. The aim of this research was to determine the appropriate amount of epidural morphine and hydromorphone needed that could provide 50% of patients with effective analgesia following hemorrhoidectomy using an up-down sequential allocation strategy, and to calculate the ratio of epidural morphine to hydromorphone administration.

## Materials and methods

### Recruitment of patients

The Ethics Committee of Dongguan Tungwah hospital approved the study protocol *(DHKY-2023-003-01)* and registered at https://www.chictr.org.cn/*(*01/03/2023) and registration number was ChiCTR2300068847. We recruited 100 patients who were hospitalized in Dongguan Tungwah hospital between March 2023 and June 2023 in accordance with the Declaration of Helsinki. Before they underwent elective hemorrhoidectomy at Dongguan Tungwah hospital, the patients were randomly recruited by one of our anesthesiologist as the research subjects, and confirmed that informed consent was obtained from all subjects and/or their legal guardian(s). Each patient was given either epidural morphine or hydromorphone using a computer-generated blocked randomization technique by a research anesthetist using SPSS for Windows version 25 (SPSS Inc., Chicago, IL, USA). At the time of randomization, sealed envelopes containing the group allocation codes were opened. The randomization procedure was carried out and the study medicine was manufactured within 60 min of administration by a blind nurse not engaged in patient consent or data collecting. Put it in another way, the study participants and all researchers were unaware of the specific study group assignments, and treatment dose.

### Subjects

The study participants fulfilled the following requirements: age of 18 to 60 years with Body Mass Index (BMI) < 30 kg/m2, American Society of Anesthesiologists (ASA) status of I-II and patients who were to undergo hemorrhoidectomy under CSEA in Dongguan Tungwah hospital. Following were the exclusion standards: allergy to the drugs (opioids and local anesthetics) used in this study, coagulation dysfunction, a history of any painkillers, alcohol, or other addictive substances, mental and neurological disorders within the previous two weeks, patients with contraindications to spinal anesthesia puncture and unwillingness to cooperate with anesthesia, and skin infections in the area to be punctured.

### Study and reference drugs

The beginning dosages of epidural hydromorphone and morphine were 0.3 mg and 1.0 mg, respectively, referring to the current literature [7, 8] and the past experience of our institution. Steps “up” were larger than steps “down” because we were concerned about insufficient analgesia (larger decreases may lead to the chance of inadequate analgesia increasing) and that we intended to identify an efficient dosage should it drop below the dose we started with (0.05 mg epidural hydromorphone and 0.1 mg epidural morphine). The dosages labeled as “up” were intended to reflect typical clinical doses commonly used in medical practice.

The potential dosage ranges for epidural hydromorphone are provided below, with the starting dose highlighted in bold:

0.1-0.15-0.2-0.25-**0.3**-0.35-0.4-0.45-0.5-0.55 mg.

The potential dosage ranges for epidural morphine are provided below, with the starting dose highlighted in bold:

0.6–0.7–0.8–0.9-**1.0**-1.1 -1.2 -1.3-1.4-1.5 mg.

A 10-piont verbal numeric rating scale (NRS) was used to assess the patients’ level of pain intensity, with 0 meaning “no pain” and 10 meaning “the most agonizing pain possible”, respectively [13]. The dosage of epidural opioids for subsequent study patients was adjusted based on the effectiveness observed in previous patients. If no rescue analgesia was required or if the NRS pain score was ≤ 3 any time point within 24 h (6, 12 and 24 h) after CSEA treatment, analgesia was considered “effective”. To estimate the ED50, doses for future patients were modified using the a sequential allocation biased-coin design technique according to the references [14–16].

If the patient has a pain score of more than 3 on the NRS after any time point within 24 h (6, 12 and 24 h) or required tramadol/parecoxib sodium rescue analgesia, The subsequent person randomly assigned to take that medication would receive a higher dose. If the verbal NRS rating for pain score were ≤ 3 at 24 any time point within 24 h (6, 12 and 24 h) or don’t need remedial analgesia, the probability that the subsequent patient will receive the same dose as the preceding patient is a 8/9 chance or a 1/9 chance of receiving a lower dose. After randomization, excluded patients were removed from our study. When a new patient was recruited, they were given either epidural morphine or hydromorphone at random and we would made adjustments to dose assignment based on the same allocation procedure. The study was terminated with 40 patients enrolled in each group.

### Study procedure

After establishing intravenous catheter in the surgical waiting areas, patients were moved into the surgery room. Pulse oxygen saturation (SPO^2^), heart rate (HR), and noninvasive blood pressure (NIBP) are continuously monitored every 5 min were used to record their initial vital signs. Spinal anesthesia drug was comprised of 0.5% bupivacaine 2 ml, and either epidural anesthesia drug of morphine (Northeast Pharmaceutical Group Review First Pharmaceutical Co., LTD., China) or hydromorphone (Yichang Renfu Pharmaceutical Co., LTD., Hubei Province, China) randomly. The preparation of the preservative-free hydromorphone and morphine was done by Dongguan Tungwah hospital anesthesiology Pharmacy. The 0.1 mg/l hydromorphone concentrations were as follows: 1 mg hydromorphone(1 mg/l) 1 ml + 9 ml 0.9%normal saline, and the 0.05 mg/l hydromorphone concentrations were as follows: 1 mg hydromorphone(1 mg/l) 1 ml + 19 ml 0.9% normal saline. The 1 mg/l morphine concentrations were as follows: 10 mg morphine(10 mg/ml) 1 ml + 9 ml 0.9% normal saline, and the 0.1 mg/l morphine concentrations were as follows: 1 mg morphine (1 mg/ml) 1 ml + 9 ml 0.9% normal saline. The unblinded nurse, who had no other clinical or research responsibilities, prepared the study drug by using a sterile technique to transfer it into a 10-mL graduated syringe separately. The research solution was then diluted by 0.9% normal saline to give each mixture a final volume of 8 ml. We used the common “one point” injection technique combined with spinal and epidural analgesia. Patients were positioned in the left side lumbar anesthesia position while receiving a 500-mL continuous intravenous infusion bolus of lactated ringer’s solution. Using ultrasound, the L3-4 or L2-3 interspace was identified as the location of the puncture space, and mark the location with a marker pen. Sterile technique was employed to clean the puncture site’s skin and lay down an aseptic treatment sheet. A 27-G pencil-tip needle was inserted, when a 16-G Tuohy needle identified through the loss-of-resistance-to-air technique. When cerebrospinal fluid flowed out, 2.0 ml of 0.5% bupivacaine was given in the subarachnoid space. After completion of the injection, the pencil-tip needle was withdrawn, and an epidural catheter was inserted 3-4 cm slowly into the epidural space and local anesthetic (3 ml 2% lidocaine) test dose was administered via the epidural catheter. The patients were then turned over, and the anesthesia plane was adjusted to T10. The patients in the experiment were treated with PPH surgery by the same surgical group or the same specialist. 10 min before the end of the operation, the epidural injection of morphine or hydromorphone (diluted to 8 ml with normal saline) was completed, and the epidural catheter was pulled out after the injection.

All patients were given a standard order set for postoperative care that included monitoring, multimodal analgesia, therapy for nausea and pruritus, and necessary emergency care. Two tablets of ibuprofen and codeine sustained-release (each tablets containing 0.2 g of ibuprofen and 13 mg of codeine phosphate) were routinely given BID after surgery to the patients. The NRS pain score was ≥ 4 points. Tramadol 100 mg and parecoxib 40 mg was also intravenously injected as needed. Granisetron (3 mg IV) and/or droperidol (2.5 mg IV) may be used as needed to treat nausea and vomiting. Itching can be treated with nalbuphine (5 mg IV three times daily, as required).

Measurements of oxygen saturation, Richmond Agitation-Sedation Scale (RASS), and respiratory rate were also taken in the specified sequence [17].

### Measurements

At each time point (6, 12 and 24 h) after CSEA, a blinded research nurse conducted patient interviews to gather all the prospective data. Patients reported the following each moment in time:

1. The NRS Pain Intensity Scale (0–10).
2. Level of nausea (0 = none, 1 = light, 2 = medium, and 3 = severe) [8].
3. The intensity of the itching (0 = none, 1 = light, 2 = medium, and 3 = severe) [8].
4. The general level of contentment with CSEA (0 = satisfied, 1 = slightly satisfied, 2 = neutral, 3 = slightly unhappy, and 4 = dissatisfied).

According to patients marking of severity, nausea and pruritus were graded. At each time point, RASS evaluated the level of sedation as well.

All patients’ baseline information was recorded, such as gender, age, height, weight, BMI, ASA rating, and all data were collected by the blinded nurses interviews with patients at 6, 12 and 24 h after spinal combined epidural anesthesia. It should be noted that patients answered the pain score at each time point (6, 12 and 24 h) in the form of a questionnaire, the need for rescue analgesia after surgery and other adverse reactions, and the follow-up of the blinded nurses on the first and second days was verified. From the patient’s electronic medical record by another a blind doctor, the following information was taken: opioid administration within 24 h, total opioid consumption, medical treatments for nausea or pruritus, RASS and hypo-ventilation episodes (respiratory rate 10 breaths every minute). The primary outcomes included the NRS ratings for pain during rest and move each time point (6, 12 and 24 h) following surgery. Secondary outcomes included patient satisfaction, use of remedial analgesics, and severity of adverse events for nausea and vomiting, skin pruritus, and the level of sedation.

### Statistical analysis

According to sample size termination criteria of the biased-coin sequential allocation method, literature and preliminary experiment research studies implied that using 20 to 40 patients would yield stable estimates of the target estimate of ED50 and ED90 [7, 14–16, 18, 19]. The purpose of this study is to observe the ED50 of epidural morphine and hydromorphone analgesia in patients undergoing hemorrhoidectomy. Considering that 10% of the patients dropped out or had difficulty placing an epidural catheter, we recruited 100 patients in our study, and 80 patients included in analysis. We carried out all analyses separately for morphine and hydromorphone. A statistical software package (IBM SPSS Statistics Inc., Chicago, IL) was used to gather and evaluate the data. The independent T-test was used to the measurement data of a normal distribution, which were expressed as mean ± SD. Counting data is expressed as frequency/percentage and tested by χ^2^. A *P* value of 0.05 or lower was regarded as statistically significant.

Study data were collected and managed using excel of Word Processing System (WPS) Office (Kingsoft) tools. Analyzing the total number of “effective” and “ineffective” responses for each dose category for each group, probit regression analysis was employed as backup and sensitivity analysis [20, 21]. Probit regression analysis was conducted using SPSS software version 25.0 (IBM SPSS Statistics Inc., Chicago, IL) to determine the ED50 values following a sequential formula. The ED50 was determined using the isotonic regression method, and it refers to the specific dose of hydromorphone and morphine used epidurally that resulted in a successful outcome (measured by NRS scores ≤ 3 cm) in 50% of the patients at 6, 12,and 24 h after the epidural administration [22, 23]. Using 2000 bootstrap replications, the bias-corrected percentile method was employed to obtain the 95% confidence interval of the isotonic regression estimator of ED50 [24, 25]. The preparation of figures was carried out with the use of GraphPad Prism version 9.5.1 (GraphPad Software Inc. San Diego, CA, USA).

## Results

**Fig. 1:**
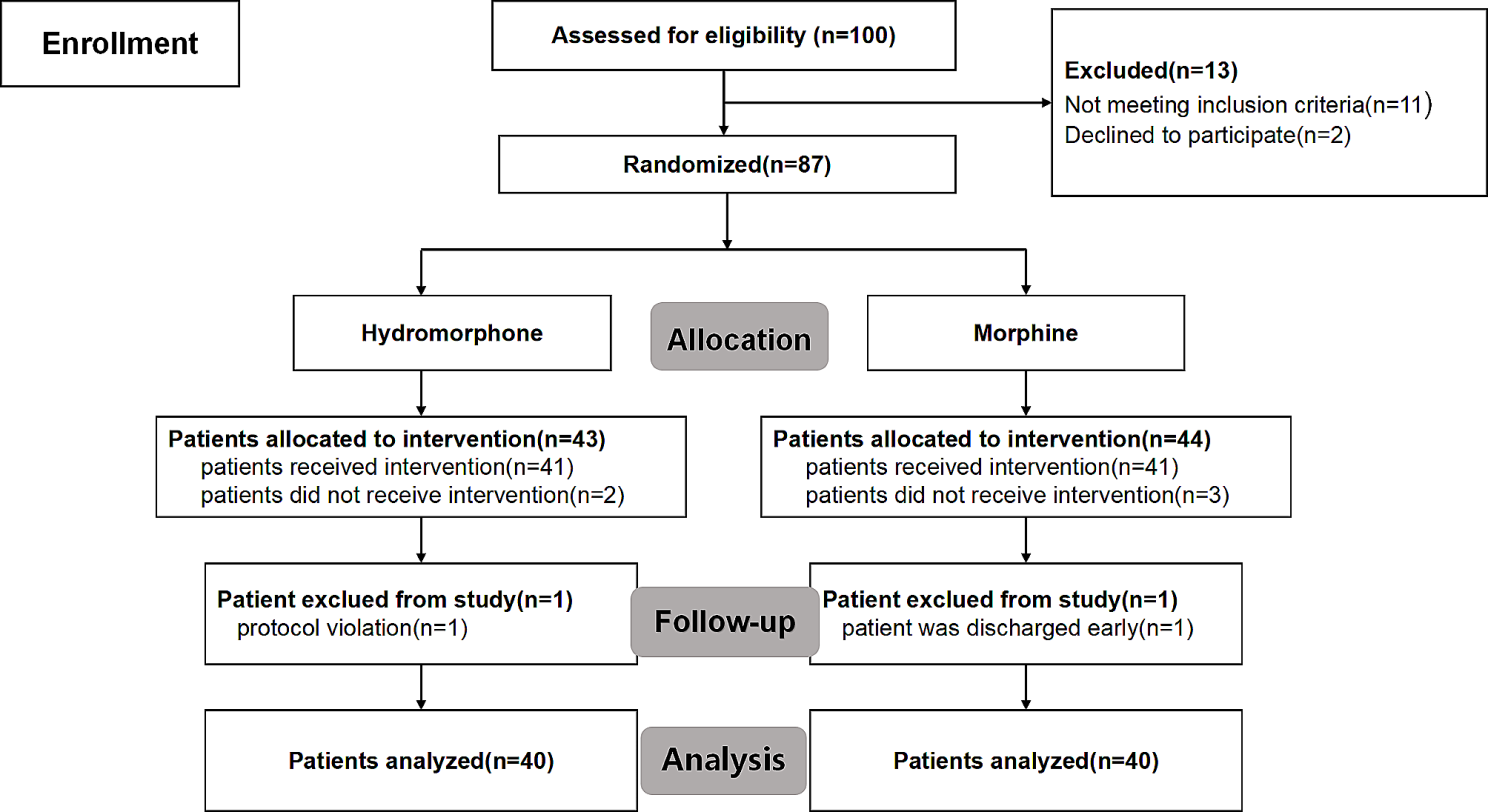
Patient assessment, randomization, allocation, follow-up, and analysis for the trial. A total of 100 patients who underwent elective haemorrhoidectomy and requested epidural combined with spinal anesthesia were included into the study. A total of 11 patients did not meet the inclusion criteria and 2 patients declined to participate, and they were excluded from the study. Epidural puncture failed in 2 patients in hydromophone group, and epidural puncture failed in 3 patients in morphine group, respectively. There were 80 patients who received the protocol analysis in two groups

From March 2023 to June 2023, 100 patients were involved in our study, and 87 participants were assigned at random to either the epidural hydromorphone group (*n* = 43) or the epidural morphine group (*n* = 44). Once the target sample size was reached, enrollment in the study came to an end. The final analysis included a total of 80 patients, with 40 patients in the epidural hydromorphone group and 40 patients in the epidural morphine group, respectively (Fig. 1). The study groups shared no significant difference in demographic and baseline characteristics (Table 1).

**Table 1:** The characteristic data

Patient characteristics	Morphine (*n* = 40)	Hydromorphone (*n* = 40)	*P* value
**Patient sex(***n***, %)**∗			*0.654*
female	20(50)	22(55)	
male	20(50)	18(45)	
**ASA physical status (***n***, %)**∗			ns
1	17(42.5)	17(42.5)	
2	23(57.5)	23(57.5)	
**Age (mean, yr)**	41.75 ± 9.16	39.53 ± 7.71	*0.244*
**Height (mean, cm)**	164.4 ± 8.17	162.4 ± 9.04	*0.308*
**Weight (mean, kg)**	61.78 ± 9.64	59.41 ± 9.00	*0.261*
**BMI (mean, kg/m** ^**2**^ **)**	22.68 ± 2.56	22.41 ± 2.55	*0.630*

Note: Data are presented as mean ± SD and frequency

Abbreviations: BMI, body mass index; ASA, American Society of Anesthesiologists status

∗Data are numbers (%)

**Fig. 2:**
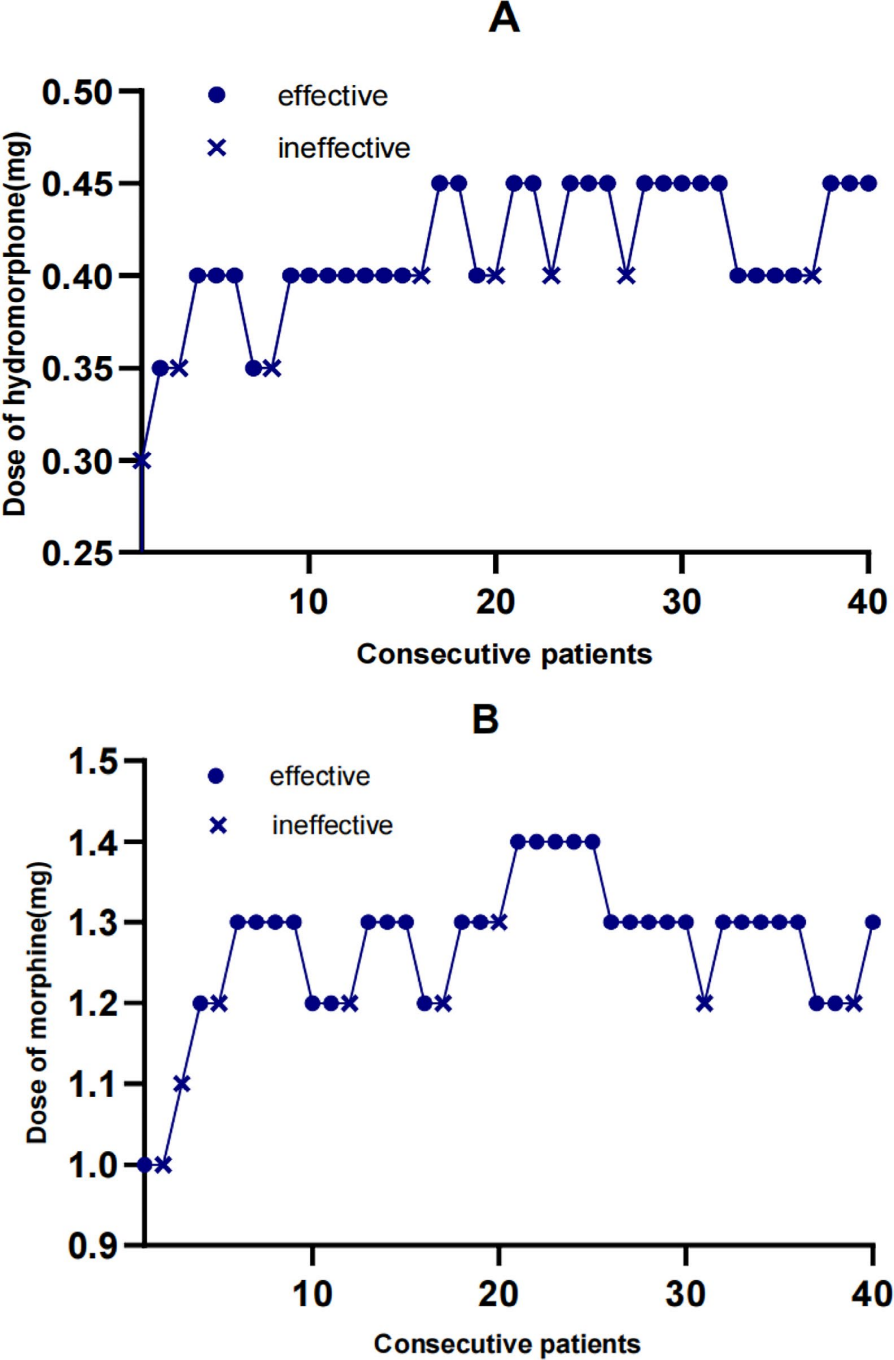
The up-down sequences of administered dose of epidural hydromorphone group (**A**) and epidural morphine group (**B**). The patient sequence number (X-axis) is the order of epidural hydromorphone (**A**) and epidural morphine exposures (**B**) using the up-down sequential allocation with a biased-coin method. The assigned dose levels are presented on Y-axis. Analgesic “successes” are represented by a point, and analgesic “failures” are represented by a fork. The ED50 was 0.350 mg (95% CI, 0.259–0.376 mg) in hydromorphone group and 1.129 mg (95% CI, 0.903–1.187 mg) in morphine group estimated using isotonic regression and bootstrapping method

The ED50 of epidural hydromorphone and epidural morphine for elective hemorrhoidectomy, using the Probit regression, was calculated to be 0.366 mg (95% CI, 0.276–0.388 mg) for epidural hydromorphone and 1.138 mg (95% CI, 0.910–1.201 mg) for epidural morphine. The ED50 was 0.350 mg (95% CI, 0.259–0.376 mg) in hydromorphone group and 1.129 mg (95% CI, 0.903–1.187 mg) in morphine group, respectively, estimated by isotonic regression and bootstrapping method (Fig. 2; Table 2). These estimates of the ED50 for hemorrhoidectomy generated a ratio of 3 (95% [CI], 3.10–3.30).

**Table 2:** Observed and PAVA-adjusted response rates

Assigned Dose, mg	No. Of Successes	No. Of Patients	Observed successful rates	PAVA-adjusted successful rates
**Morphine**				
1	1	2	0.500	0.250
1.1	0	1	0.000	0.250
1.2	6	11	0.540	0.540
1.3	20	21	0.952	0.952
1.4	5	5	1.000	1.000
**Hydromorphone**				
0.3	0	1	0.000	0.000
0.35	2	4	0.500	0.500
0.4	15	20	0.750	0.750
0.45	15	15	1.000	1.000

PAVA = pooled-adjacent-violators algorithm

∗PAVA-adjusted response rates were estimated using the weighted isotonic regression method

Table 3 provides a summary of side effect occurrence, treatment, and general satisfaction. Vomiting, nausea, sedation, pruritis, or any other opioid-related adverse effects were not significantly different across study groups following surgery. The occurrence rates were 5% (2/40) for nausea, 7.5% (3/40) for vomiting and 10% (4/40) for pruritus over the first 24 h in the hydromorphone group, respectively. The occurrence rates were 2.5% (1/40) for nausea, 5% (2/40) for vomiting and 15% (6/40) for pruritus over the first 24 h in the morphine group, respectively (Table 2). Patients didn’t need additional medicine because the severity of the patients’ nausea, vomiting, and pruritus was mild in both groups. There were no cases of respiratory depression. We found that all patients did not have any serious adverse reaction.

**Table 3:** Incidence of nausea or vomit, pruritus and patients’ satisfaction degree

Group (*n*,%)	Nausea	Vomiting	Pruritus	Unsatisfactory analgesic effect	Satisfactory analgesic effect
**Morphine (** *n* ** = 40)**	1(2.5)	2(5)	6(15)	1(2.5)	39(97.5)
**Hydromorphone (** *n* ** = 40)**	2(5)	3(7.5)	4(10)	1(2.5)	39(97.5)

Date are presented as *n*(%)

Both epidural morphine and epidural hydromorphone were well-recognized. Independent of dosage, 97.5% (39/40) and 97.5% (39/40) of patients, respectively, reported to be “satisfied” 24 h after hemorrhoidectomy with their overall pain control (Table 3).

## Discussion

The ED50 of epidural hydromorphone and epidural morphine for hemorrhoidectomy were 0.366 mg (95% [CI], 0.276–0.388 mg) and 1.138 mg (95% [CI], 0.910–1.201 mg), respectively, which follows a prospective, double-blinded, randomized up-down sequential allocation design. There is a calculated ratio that estimates the strength comparison of epidural morphine to hydromorphone was 3:1 for hemorrhoidectomy analgesia. There are no significant differences found among the epidural hydromorphone group and the epidural morphine group in terms of ongoing opioid usage, patient satisfaction, and the frequency and severity of side effects, according to the exploratory studies.

Opioids are widely used therapy for pain during the postoperative period. Some studies suggest that hydromorphone is clinically superior [26]. Hydromorphone is commonly prescribed as an alternative for the management of moderate to severe pain instead of morphine. Although hydromorphone has been studied thoroughly from the perspective of cancer pain [27], chronic noncancer pain, and cesarean delivery [7–9]. Satisfactory pain relief with reduced consumption of sufentanil can be achieved by administering 0.6 mg of hydromorphone alongside ropivacaine through an epidural after a cesarean section [28]. Puhto et al. [29] found that the distribution and elimination of hydromorphone after a single epidural dose appears to be comparable to what has been documented for individuals who are not pregnant following intravenous administration of hydromorphone. However, the Puhto et al. study is limited by seven healthy parturients who only receive dose of 1.5 mg, 0.75 mg and 0.5 mg. In our study design, we seek to understand the associations between dose of epidural hydromorphone and the postoperative pain of hemorrhoidectomy, and there is allowance for the administration of a broad spectrum of doses.

An oral morphine to oral hydromorphone conversion ratio of 8:1 mg/mg is employed for patients experiencing ongoing moderate to severe pain associated with cancer or non-cancer conditions [30]. Intravenous administration of morphine and hydromorphone have been at around a 5:1 potency ratio [8, 31, 32]. Sviggum et al. have found that [7], the ideal dosage for intrathecal morphine and intrathecal hydromorphone to be given for cesarean-section analgesia is 2:1. The current research obtains a highly important finding by determining that the potency ratio of epidural morphine to epidural hydromorphone is 3:1. However, the use of incremental dose steps in the sequential allocation method introduces some uncertainty in calculating the ED50. This new finding has clinically significance since it will help anesthesiologists decide how much of these two opioids to provide via epidural, so as to achieve more accurate dose conversions.

While epidural opioids have some side effects such pruritus, nauseousness, vomiting, sedation, and respiratory depression, they are useful at managing pain. According to a randomized crossover study conducted on healthy volunteers, there is evidence to suggest that hydromorphone could possess a more favorable clinical profile in terms of its effects, side effects, and variability when compared to morphine [33]. An observational study, using a prospective case-matched design, was conducted to assess elective surgery patients, and it revealed that there were minimal statistical discrepancies in terms of major adverse effects between epidural fentanyl and epidural hydromorphone [34]. This study includes a total of 119 women who gave birth. The results show that when esketamine is added to morphine, it enhances the pain relief experienced after cesarean section without causing more negative effects [35]. Our study findings are consistent with previous research, as there is no variance in the occurrence of nausea, vomiting, and pruritus between the groups.

It is important to mention that the purpose of up-down biased-coin studies is not to compare the negative effects of epidural hydromorphone with morphine [7]. The study is intended for determining the appropriate dosage, and the ED50 for hemorrhoidectomy. Also, opioids can cause severe sedation and respiratory depression, even if they are uncommon. In this trial, there are no reports of severe sedation and related respiratory depression at any dose. Nonetheless, the sample size was inadequate to draw any firm conclusions about the relative safety of the two medications.

To minimize the number of subjects involved and resources utilized during a trial, certain studies employ up-down study designs with varying stopping rules in a relatively small sample of patients (around 20–40) [20, 36, 37]. Similar to earlier anesthesiology investigations, we adopt an up-down stopping rule with a predetermined sample size. Our goal is to collect exploratory data on all subjects, ensuring that the oversampling does not pose a clinical risk to the patients involved in our study.

There are still some limitations in the current study. First, the dose of bupivacaine was 0.5% 2 ml in all patients, causing a difference in the analgesic effectiveness between each patient. Second, the time point of the data is controversial because the analgesic effect is defined by the verbal numerical rating scale score for pain within 24 h. Nevertheless, this does not influence the results of ED50 epidural hydromorphone/morphine for hemorrhoidectomy. Third, the data comes from one region, and the population is homogeneous. These findings may not be applicable to other populations. Furthermore, it is important to mention that while the patients were randomly assigned to either the epidural hydromorphone or morphine groups, the focus of this study was not to assess the analgesic effectiveness between the two groups. Fourth, this study is not included the patient’s blood pressure and heart rate within 24 h after dosing. The main purpose of our study was to determine the appropriate amount of epidural morphine and hydromorphone needed that could provide 50% of patients with effective analgesia following hemorrhoidectomy using an up-down sequential allocation strategy. At the same time, and may consider that the main side effects of hydromorphone and morphine are nausea and vomiting, pruritus, etc [7]. More relevant research is needed to determine if hydromorphone has a comparable duration of pain relief as morphine and to evaluate the adverse effects of both drugs when given at equivalent doses in different population.

## Conclusion

In brief, the up-down sequential allocation method was used to estimate that the potency ratio of epidural morphine to hydromorphone for pain relief after hemorrhoidectomy is 3:1. The ED50 for epidural morphine analgesia within 24 h after hemorrhoidectomy is calculated to be 1.138 mg, while the ED50 for epidural hydromorphone is 0.366 mg. The exploratory analysis reveals a high level of patient satisfaction with both drugs and it appears that there is no noticeable difference in the negative side effects of either drug at the given doses. Ultimately, the results are expected to offer guidance in choosing between morphine or hydromorphone for providing pain relief after an acute hemorrhoidectomy in multimodal analgesia.

### Electronic supplementary material

Below is the link to the electronic supplementary material.

Supplementary Material 1

## Data Availability

The datasets used and/or analysised during the current study are available from the corresponding author on reasonable request.

## Abbreviations

ED50: Effective analgesic dose for 50% patients
CSEA: Combined spinal and epidural anesthesia
BMI: Body Mass Index
ASA: American Society of Anesthesiologists
NRS: Numeric rating scale
SPO^2^: Pulse oxygen saturation
HR: Heart rate
NIBP: Noninvasive blood pressure
RASS: Richmond Agitation-Sedation Scale
