# Supplementary material for: The 50% effective dose of hydromorphone and morphine for epidural analgesia in the hemorrhoidectomy: a double-blind, sequential dose-finding study
Source: BMC Anesthesiol. 2024 Jan 30;24:41. doi: 10.1186/s12871-024-02420-0 (PMC10826036; doi:10.1186/s12871-024-02420-0)
Supplement: Supplementary file 1 — Supplementary Material 1 [file 12871_2024_2420_MOESM1_ESM.docx]

**Figure 1.** **Patient assessment, randomization, allocation, follow-up, and analysis for the trial.**

A total of 100 patients who underwent elective haemorrhoidectomy and requested epidural combined with spinal anesthesia were included into the study. A total of 11 patients did not meet the inclusion criteria and 2 patients declined to participate, and they were excluded from the study. Epidural puncture failed in 2 patients in hydromophone group, and epidural puncture failed in 3 patients in mophine group, respectively. There were 80 patients who received the protocol analysis in two groups.

**Figure 2. The up-down sequences of administered dose of epidural hydromorphone group(Fig.2A) and epidural morphine group(Fig.2B)**

The patient sequence number (X-axis) is the order of epidural hydromorphone (Fig.2A) and epidural morphine exposures (Fig.2B) using the up-down sequential allocation with a biased-coin method. The assigned dose levels are presented on Y-axis. Analgesic “successes” are represented by a point, and analgesic “failures” are represented by a fork.The ED50 was 0.350 mg (95% CI, 0.259–0.376 mg) in hydromorphone group and 1.129 mg (95% CI, 0.903–1.187 mg) in morphine group estimated using isotonic regression and bootstrapping method.

**Table 1.** **The Characteristic Data**

**Table 2.** **Observed and PAVA-adjusted response rates**

**Table 3. Incidence of Nausea or Vomit, Prurits and Patients’ satisfaction degree**
